# Amygdala temporal dynamics: temperamental differences in the timing of amygdala response to familiar and novel faces

**DOI:** 10.1186/1471-2202-10-145

**Published:** 2009-12-10

**Authors:** Jennifer U Blackford, Suzanne N Avery, Richard C Shelton, David H Zald

**Affiliations:** 1 Department of Psychiatry, Vanderbilt University Medical School, Nashville, Tennessee, USA; 2 Department of Psychology, Vanderbilt University, Nashville, Tennessee, USA

## Abstract

**Background:**

Inhibited temperament - the predisposition to respond to new people, places or things with wariness or avoidance behaviors - is associated with increased risk for social anxiety disorder and major depression. Although the magnitude of the amygdala's response to novelty has been identified as a neural substrate of inhibited temperament, there may also be differences in temporal dynamics (latency, duration, and peak). We hypothesized that persons with inhibited temperament would have faster responses to novel relative to familiar neutral faces compared to persons with uninhibited temperament. We used event-related functional magnetic resonance imaging to measure the temporal dynamics of the blood oxygen level dependent (BOLD) response to both novel and familiar neutral faces in participants with inhibited or uninhibited temperament.

**Results:**

Inhibited participants had faster amygdala responses to novel compared with familiar faces, and both longer and greater amygdala response to all faces. There were no differences in peak response.

**Conclusion:**

Faster amygdala response to novelty may reflect a computational bias that leads to greater neophobic responses and represents a mechanism for the development of social anxiety.

## Background

Temperament refers to stable patterns of emotions, thoughts and behaviors, which are observable in early childhood and appear to be biologically-based. Most models of temperament include a construct related to distress and avoidance [e.g., [1-5]]. The temperament construct of behavioral inhibition, or "inhibited temperament", is defined as the predisposition for a person to respond to new people, places or things (i.e., novelty) with wariness or avoidance behaviors [3]. Inhibited individuals are likely to show wary, avoidant, or fearful responses to novelty. In contrast, uninhibited individuals typically respond to new people and things with positive approach behaviors. Inhibited temperament has a well-characterized phenotype and is associated with increased risk for both social anxiety disorder [6,7] and major depression [8]. The study of inhibited temperament may thus provide clues about a specific developmental trajectory for anxiety and depressive disorders.

Based on its reactivity to novelty and other types of uncertainty, individual differences in amygdala functioning have been proposed as a neural substrate of inhibited temperament [9]. The amygdala's response to faces may be particularly salient for inhibited temperament, given the heightened risk for social anxiety. Face stimuli strongly elicit amygdala responses [10,11], with several studies suggesting the response is modulated by individual differences in inhibited temperament [12-14]. For example, Schwartz and colleagues [12] found that young adults who had been identified as behaviorally inhibited during childhood showed greater amygdala activation to novel relative to newly familiar faces, compared to those who were behaviorally uninhibited. These results support the involvement of the amygdala in mediating temperamental differences, but do not address whether there are any differences in the temporal dynamics, or *timing*, of the amygdala response, such as latency, duration, and peak.

Individual differences in the temporal dynamics of emotional response, or "affective chronometry", have been suggested as an important component of affective style [15]; for example, a typical pattern of emotional response might be characterized as a "quick temper" or being able to "recover quickly" from negative emotions. Differences in the time course of behavioral affective responses have been associated with individual differences in both introversion/extraversion [16] and depression [16,17]. These individual differences in the time course of affective responses presumably reflect differences in the temporal dynamics of brain regions supporting emotional responses, such as the amygdala.

We are not aware of any studies of temperamental differences in the temporal dynamics of amygdala response, although a finding from the anxiety literature highlights the potential importance of studying temporal dynamics. Amygdala latency and magnitude for people with a spider phobia were compared to non-phobic participants in an event-related fMRI study [18]. Participants viewed pictures of spiders and neutral pictures. Spider phobics had shorter latencies to respond to the pictures of spiders (compared to neutral) than the nonphobic participants, even though the magnitude of the amygdala's activation did not differ between the two groups. Similar to spider phobics, persons with inhibited temperament can be considered to have neophobia, or a fear of novelty. Neophobic responses are not necessarily identical to simple phobic responses, as they are both more generalized and often weaker in intensity, but they nevertheless involve clear perception of certain stimuli as potentially threatening, and engender avoidance responses. To the extent that these phobias reflect a similar neurobiology, persons with inhibited temperament may be predicted to have faster amygdala responses to novel stimuli.

In this study, we used a slow event-related fMRI paradigm to measure the temporal dynamics--latency, duration, and peak--of amygdala response to novel neutral faces compared to newly familiarized neutral faces in persons with inhibited or uninhibited temperament. The event-related paradigm allows estimation of the temporal dynamics of the blood oxygen level dependent (BOLD) signal in a specific region. We hypothesized that inhibited participants would have an amygdala response characterized by shorter latency when viewing novel compared to familiar neutral faces. Given that magnitude differences have been observed in past studies of inhibited [12] individuals and that latency differences alone could not account for these differences, we hypothesized that either the peak and/or the duration of the activations would be enhanced in inhibited individuals.

## Results

### Behavioral Data

To determine if there were temperamental differences in face memory, we compared performance on immediate and delayed face memory. On the immediate memory task, average performance was at the 50^th ^percentile (scale score = 10) for both the inhibited (*M *= 10.5, *SD *= 3.1) and uninhibited (*M *= 10.1, *SD *= 2.3) groups, *t*(18) = .33, *p *= .74. Delayed memory performance was similar for both the inhibited (*M *= 10.2, *SD *= 3.2) and uninhibited (*M *= 11.6, *SD *= 3.2) groups, *t*(18) = -.98, *p *= .34.

To validate that participants were actively engaged in the task and to assess possible group differences in memory for the faces shown during the task, we performed a post-scan recognition task using both examples of the familiar and novel faces from the task. Both groups demonstrated similar accuracy for both the familiar faces (inhibited = 98%; uninhibited = 98%) and the novel faces (inhibited = 82%, uninhibited = 81%).

### Temporal Dynamics

To determine if the temperament groups differed in the timing of the amygdala's response to faces, we compared onset latency, duration, and peak of response to novel relative to familiar faces. For each analysis, we used a repeated measures analysis of variance with temperament group (inhibited/uninhibited) as the between-subjects factor and face type (novel/familiar) as the within-subjects factor.

#### Latency

**Figure 1:**
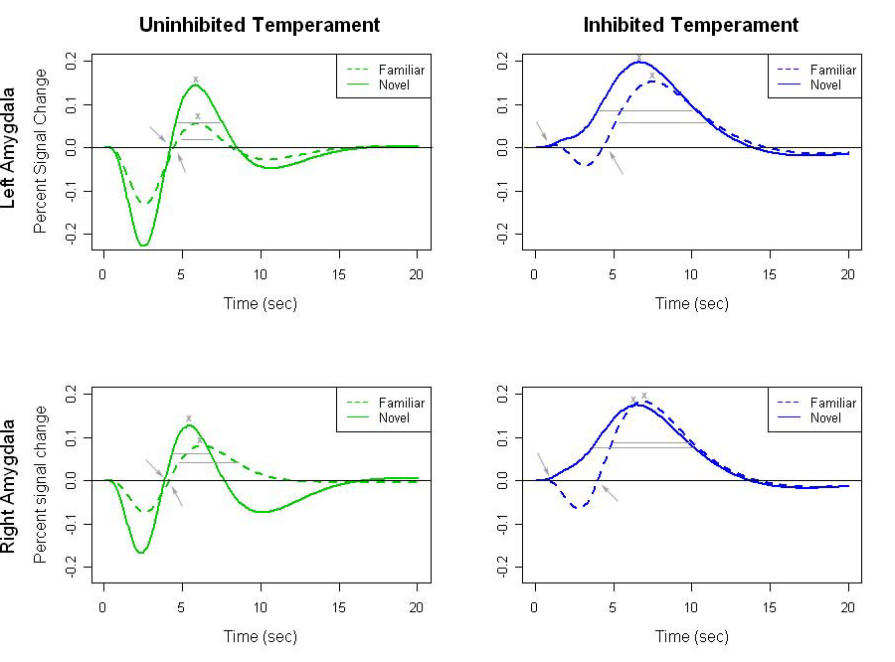
**Inhibited participants respond more quickly to novel compared to familiar faces**. Fitted time courses for left and right amygdala by temperament group (inhibited/uninhibited) and face type (novel/familiar) illustrate differences in temporal dynamics. Inhibited participants had a faster amygdala response to novel relative to familiar faces in the left and right amygdala (onset marked by arrows). The duration of the amygdala response (marked by horizontal lines) to both novel and familiar faces was longer in the inhibited group. Peak response (indicated by x) failed to differ significantly between groups.

Temperament group differences emerged in the temporal dynamics of amygdala activations. For the onset latency, there was a significant interaction of temperament group and face type in the left amygdala, *F*(1,16) = 8.13, *p *= .01, and in the right amygdala, *F*(1,16) = 9.70, *p *= .01 (see Figure 1). There were no significant main effects. We followed the significant interaction findings with post-hoc analyses. In the right amygdala, the interaction reflected a significantly faster onset to novel compared to familiar faces in the inhibited group, F(1,7) = 7.90, *p *= .03, but not the uninhibited group (*p *= .53). In the left amygdala, the effect of face type did not reach our statistical threshold in either group, but there were trends toward a faster onset to novel faces in the inhibited group (*p *= .09) and a faster onset to familiar faces in the uninhibited group (*p *= .13).

#### Duration

The duration of the amygdala's response to all faces differed by temperament group with inhibited participants showing a longer duration of response to both familiar and novel faces (see Figure 1). The main effect of temperament group was significant for both the left and right amygdala, *F*(1,16) = 6.98, *p *= .02 and *F*(1,16) = 4.73, *p *= .05, respectively. There were no other significant effects for either amygdala, indicating that duration reflects a general difference in temporal dynamics that is not specific to novel stimuli.

#### Peak

Peak amygdala response did not differ significantly across temperament groups, face types, or the interaction of Temperament Group × Face Type.

### Magnitude

**Figure 2:**
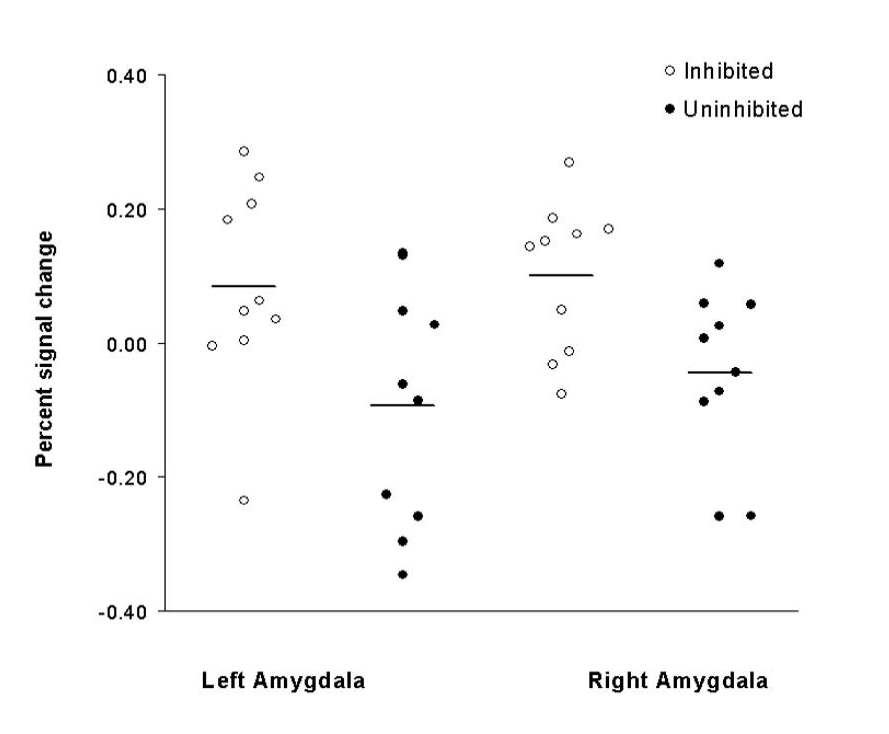
**Inhibited participants have greater magnitude of amygdala response to faces**. Dot plots of mean percent signal change to all faces (novel and familiar) for the inhibited and uninhibited temperament groups. The difference in magnitude of amygdala response between groups was significant in the right amygdala (*p *= .02).

Magnitude represents the overall amygdala response as typically measured in fMRI studies. To assess whether the inhibited participants showed a greater amygdala response to novel faces, we compared magnitude of amygdala response to novel compared to familiar faces between the two temperament groups. For magnitude of amygdala response, there was a main effect of temperament group in the right amygdala, *F*(1,16) = 7.38, *p *= .02, with response to faces greater in the inhibited temperament group (see Figure 2). In the left amygdala, the magnitude was also larger in the inhibited group, but the difference failed to reach significance (*p *= .11). We did not observe a Temperament Group × Face Type interaction, as the inhibited group showed enhanced responses to both novel and familiar stimuli, rather than a selective increase in the magnitude of response to novel relative to familiar faces.

### Whole-brain analysis

**Figure 3:**
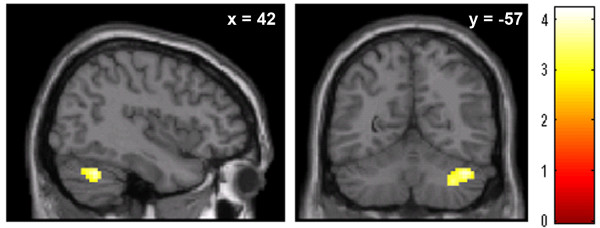
**Inhibited participants show greater BOLD signal to novel faces in cerebellum**. When viewing novel compared to familiar faces, persons with inhibited temperament demonstrated significantly stronger BOLD signal in the right cerebellum (Crus I, lobule VI). Activation maps are superimposed on sagittal (left image) and axial (right image) sections of a single standard brain image (MNI canonical T1 image). Maps are thresholded at voxel *p *< .005 and contiguous cluster size > 40, with the color bar representing *t*-values.

To determine if other brain regions showed a response to the novel faces, we performed exploratory whole brain analyses testing for temperament differences of the novel > familiar contrast. The inhibited participants showed a greater BOLD response for the novel > familiar contrast in the right cerebellum (see Figure 3). The cerebellar cluster (peak voxel: *z *= 3.47, *p *= .001; x = 33, y = -66, z = -27; cluster size = 127) included both Crus I and lobule VI. There were no brain regions where BOLD response was greater for the uninhibited participants for the novel > familiar contrast. While overall activation to faces, across subjects, was seen in expected regions, such as fusiform face area, thalamus, and visual cortex, none of these differed significantly by temperament group.

## Discussion

The results of our study provide initial evidence for temperamental differences in the temporal dynamics, or timing, of the amygdala's response to faces. The amygdala of inhibited persons responded more quickly to novel relative to familiar faces and had both longer and greater magnitude of amygdala response to both novel and familiar faces.

These results extend previous reports of temperament-based differences in magnitude of amygdala response [12-14] by demonstrating temperament-related differences in the amygdala's latency to respond to novel faces. Our observation of shorter response latency in inhibited temperament is consistent with a previous study showing amygdala latency differences in spider phobics viewing spider versus neutral pictures [18]. Both studies found the latency difference in the entire amygdala region of interest. Group differences in the amygdala response were only detectable because latency differences were specifically modeled and examined, demonstrating the importance of measuring temporal dynamics. Faster amygdala responses may reflect a bias for detecting novelty and potential threat [19,20]. This bias could result from either a higher saliency evaluation (leading to a shorter time to reach a threshold of processing), or through a more generally lowered threshold (such that even modestly salient or ambiguous stimuli can engage amygdala processes). In either case, the more rapid engagement of the amygdala processing may lead to faster triggering of limbic mediated or modulated processes, including heightened orienting responses and vigilance for potential threat.

Persons with inhibited temperament had longer amygdala responses to both novel and familiar faces suggesting a general difference in temporal dynamics that was not specific to novelty. This finding stands in contrast to modeling of spider phobic responses, in which Larson et al. [18] observed shorter duration responses. The shortened duration in that study may have been caused by a visual avoidance of the spider pictures by the spider phobics, whereas in this study, the pictures of neutral faces were unlikely to elicit a strong avoidance response, even for people with social anxiety. The longer duration in the present study suggests a prolonged engagement of the amygdala. Given the ambiguous nature of novel social stimuli, a longer amygdala response could reflect a prolonged appraisal period. Alternatively, the prolonged activity may also reflect an extended influence of other brain regions, such as the visual cortex, or less effective inhibition of the amygdala by prefrontal cortical regions involved in regulating limbic responses. Future effective connectivity analysis might advance our understanding of temperamental differences in the relationships between visual cortex, prefrontal cortex, and the amygdala.

Inhibited participants had a greater magnitude of amygdala response to both familiar and novel faces suggesting a generalized response to faces. Beaton and colleagues [14] reported a similar finding in their study of amygdala responses to faces of strangers compared to friends in people classified as shy or bold (a construct related to inhibited temperament). However, this general response to faces is not consistent with the selectively increased response to novel versus familiar faces initially reported by Schwartz and colleagues [12]. Differences in either study design or samples may account for the discrepancy. First, Schwartz used a block design whereas both Beaton and we used an event-related design. The alternating novel-familiar block design used by Schwartz and colleagues inherently provides paradigmatic familiarity: once a participant sees the first stimulus in a block, they know they will see that same stimuli repeatedly and for alternating blocks, they also know what the next block will be. In contrast, studies with random presentation of the conditions (like our event-related design) prevent participants from knowing what they will see next (and when). This may increase uncertainty about, and therefore amygdala response to, even the familiar faces. Also, because block designs provide greater statistical power to detect changes in BOLD signal, the failure to find the novelty effect may be a Type II error. Second, Schwartz selected adult participants based on behavioral observations made during childhood. Beaton used a current self-report measure in adults, and we used both current and retrospective self-report in adults. The identification of inhibited temperament in childhood may have contributed to the greater amygdala response to novelty as the early assessments may more closely reflect the underlying biology, prior to the influence of environment. Moreover, the childhood assessment in the Schwartz et al. study was based on a direct assessment of behavioral responses to multiple types of novelty, which may further heighten the ability to observe differential biological responses to novelty in their sample.

Increased activation to novel faces in inhibited persons was evident in the right cerebellum. The cerebellum has traditionally been viewed as controlling motor function; however, evidence is accumulating for a broader preparatory function in multiple neural systems, including sensory, attention, and memory systems (for a review see [21]). Temperamental differences in the novelty responses of the cerebellum may reflect heightened preparatory responses to sensory stimuli, particularly potentially salient visual stimuli such as novel human faces.

Several caveats are warranted in interpreting the findings from the present study. First, we used self-report measures to assess both childhood and current inhibited temperament. Researchers in the field of inhibited temperament (behavioral inhibition) have traditionally relied on behavioral assessments of temperament during infancy or early childhood. Early behavioral assessments may be ideal for identifying biological-based differences which are yet to be significantly impacted by environment. However, these longitudinal, prospective studies are not practical for most researchers. The self-report instruments used here were developed for consistency with the infant and toddler behavioral assessments, have good reliability and validity, and have been associated with expected outcomes like social anxiety [22]. Second, the sample size for the study was relatively small. However, amygdala responses have been consistently demonstrated with samples of this size, including the study by Schwartz and colleagues [12], and were large enough to see differences in temporal dynamics and overall duration and magnitude. Post-hoc power analysis suggest that a sample size twice as large may have detected additional main effects of temperament, but no other novelty effects. Still, given the reduced statistical power afforded by small sample sizes, these results should be interpreted with appropriate caution. Finally, there was a trend for ethnicity to differ between the two temperament groups. To ensure that ethnicity did not confound the temperament results presented, we performed post-hoc tests for ethnicity effects within the inhibited group and found no significant differences.

## Conclusion

Although fMRI studies have increasingly explored potential neural correlates of personality, the timing of the brain's response has been almost completely ignored. Findings from this study provide initial evidence for temperamental differences in the temporal dynamics of the amygdala's response to novel and familiar faces. Extending our knowledge to individual differences in how the brain responds to stimuli may provide new avenues for identifying underlying mechanisms of temperamental risk for the development of anxiety and depressive disorders.

## Methods

### Participants

Twenty persons participated in the study based on having very inhibited (n = 10) or uninhibited (n = 10) temperament in both childhood and adulthood. The participants had mean age of 21.7 years (SD = 3 years), were approximately half female (60%), predominantly right-handed [90%; [23]], and represented several ethnic groups. The inhibited and uninhibited temperament groups were significantly different on both the childhood and adult measures of inhibited temperament, but did not significantly differ in age, gender, or handedness (Table 1). There was a trend for a group difference in the distribution of ethnicity (*p *= .07). The inhibited group had a higher proportion of Asian-Americans, consistent with published ethnic differences in rates of inhibited and uninhibited temperament [24]. To control for potential effects due to ethnicity, we included ethnicity as a covariate in all statistical analyses.

**Table 1:** Participant Characteristics by Temperament Group

	Inhibited Temperament		Uninhibited Temperament		*p *value
	**Mean**	**SD**	**Mean**	**SD**	
		
**Temperament**					
Retrospective	3.0	.32	1.5	.22	.0001
Current	3.1	.55	1.7	.14	.0001
**Demographics**					
Age	22.20	3.62	21.30	2.45	NS
Gender (% Male)	40%		40%		NS
Handedness (% Right)	80%		100%		NS
Ethnicity					.07
% Caucasian	50%		70%		
% African-American	10%		30%		
% Asian	40%		0%		

The study was approved by the Vanderbilt University Institutional Review Board and written informed consent was obtained after participants received a complete description of the study.

### Participant Selection

Participants were recruited into a larger study of temperament using advertisements and multiple research participant databases seeking people who were "especially shy or outgoing as a child". Potential participants (N = 84) were prescreened using 11 questions about their behavior as a child and as an adult. Based on their answers, approximately half (n = 47; 56%) of the people were invited to participate, with the majority (n = 40; 85%) enrolling in a larger study of temperament. Childhood inhibited temperament was assessed using the Retrospective Self-Report of Inhibition [RSRI; [22]] which consists of 30 questions scored on a five-point scale with five representing extreme inhibition. A related measure, the Current Self-Report of Inhibition [CSRI; [22]] assessed adulthood inhibited temperament. The CSRI consists of 31 questions, also on a five-point scale. Both the RSRI and CSRI have good reliability and validity[22] and internal consistency (Cronbach's α) in this sample was high (RSRI = .94; CSRI = .95). Average scores on both the RSRI and CSRI were computed for each participant. Cutoff score guidelines (inhibited ≥ 2.6, uninhibited ≤ 2.0) were set to select approximately the top and bottom 15% of the population based on available normative data [22].

Participants for the fMRI study were selected based on: a) having extreme scores on both the RSRI and CSRI; and b) not meeting any of the fMRI exclusion criteria (use of psychiatric medications; recent history of substance abuse; serious neurological or medical disorders; history or brain injury or significant loss of consciousness; or failure on MRI safety screen). Participants were not excluded for presence or history of psychiatric illness because both inhibited and uninhibited temperament are associated with increased rates of internalizing and externalizing disorders, respectively [25].

Psychiatric history was assessed according to the Diagnostic and Statistical Manual of Mental Disorders with a Structured Clinical Interview for DSM-IV [SCID; [26]]. Interviews were conducted by a trained clinical interviewer blind to temperament group. As expected, the inhibited temperament group had increased rates of internalizing disorders compared to the uninhibited group. Specifically, four of the inhibited participants met criteria for an anxiety disorder (two Social Anxiety Disorder, one Generalized Anxiety Disorder, one Anxiety NOS), with two having a comorbid depressive disorder. None of the uninhibited participants met criteria for a current psychiatric disorder.

To provide a behavioral measure of face memory, we assessed immediate and delayed face memory using the Wechsler Memory for Faces tests [27].

### fMRI Task

#### Stimuli and Procedure

Pictures of novel and newly familiarized neutral faces were presented to participants in the scanner using Eprime software (Version 1.1, Psychology Software Tools, Pittsburgh, PA). Face stimuli were black and white human face images with neutral expressions, selected from two standard sets of emotional expressions [28,29]. All stimuli were edited to ensure uniform face size, eye position, and vertical nose bridge position. Extraneous features such as hair and shirt collars were standardized to ensure similarity across the two sets. Stimuli were randomly selected for the novel or familiar group, balanced across gender and stimulus set. Luminance values were similar across the novel and familiar groups of images.

The fMRI protocol was divided into familiarization and test phases. While in the scanner, participants were first familiarized to a set of six faces using the procedure from the Schwartz et al. study [12]. Each face was randomly presented 16 times for 0.5 second with a 0.5 second interstimulus interval (96 seconds total). The test phase consisted of three separate 348 second runs consisting of 12 novel and 12 familiar randomly presented faces each, for a total of 36 novel and 36 familiar face presentations. Each of the six familiar faces was randomly presented twice within each run, whereas the novel faces were only presented once across all three runs. Faces were presented for 0.5 second, with each presentation preceded by a 1 second fixation cross. The inter-stimulus interval was jittered (*M *= 14 seconds) to sample across multiple parts of the hemodynamic response. This sampling strategy provides a more accurate estimation of the temporal dynamics measures. Following the fMRI procedure, participants viewed 26 randomly presented faces, comprised of faces previously seen in the scanner (six familiar faces and 20 faces that were seen once), and were asked to determine whether each face was seen once or many times before.

#### MRI data acquisition

Anatomical and echo planar imaging (EPI) images were collected on a 3 Tesla Phillips Achieva magnet (Philips Healthcare, Inc., Best, The Netherlands). High resolution T1-weighted anatomical images were collected (256 mm FOV, 170 slices, 1 mm, 0 mm gap). EPI images were acquired using a sequence optimized for the amygdala: 2 s TR, 22 ms TE; 90° flip angle; 240 mm FOV; 3 × 3 mm in plane resolution using an 80 × 80 matrix (reconstructed to 128 × 128), and higher-order shimming to limit susceptibility artifacts. Each volume comprised 30 2.5 mm (.25 gap) axial oblique slices (titled 15° anterior higher than posterior relative to the intercommissural plane) which provided complete anterior-posterior coverage and inferior-superior coverage from the bottom of the temporal lobe to the top of the most dorsal part of the cingulate gyrus. For each participant, EPI images were visually inspected for artifacts and signal dropout prior to analysis to ensure appropriate coverage of the amygdala region of interest.

#### MRI data processing

MRI data were pre-processed using SPM5 http://www.fil.ion.ucl.ac.uk/ and Matlab (Version 7.1, The MathWorks, Inc, Natick, MA). Data were slice time corrected, realigned to the first slice, resampled to 3 × 3 × 3 mm voxels, spatially normalized into standard stereotactic space (MNI EPI template), and high pass filtered (128 s). Data were smoothed with an 8 mm FWHM Gaussian kernel to account for individual differences in brain anatomy.

The participant-specific general linear model [30] was estimated using both face types (novel and familiar) as regressors. Temporal (latency) and dispersion (duration) derivatives were also included in the model to provide a more precise estimate of the event-related response curve [31,32].

### Data Analysis

#### Behavioral Data

Group differences in the demographic and behavioral data were tested using *t*-tests for continuous variables and chi-square analysis for categorical variables (alpha = .05). We used SAS statistical software (Version 9.1, SAS Institute Inc., Cary, NC) to perform the analysis.

#### Temporal Dynamics

The bilateral amygdala regions of interest (ROI) were defined using the anatomical amygdala templates based on Automated Anatomical Labeling [AAL; [33]] implemented in the WFU Pick Atlas [Version 2.4; [34]]. For each participant, we extracted fitted response curves for the novel and familiar face conditions using MarsBar [35]. For each response curve, latency was computed as the onset of a reliable increase (> .01 percent signal change) in the hemodynamic response function. Duration was measured using the full width half maximum value (FWHM), measured as the distance between two points on the response curve where the function reaches half of the peak value. Amygdala latency and duration values were log transformed prior to analysis to normalize their distributions. Peak was defined as the largest value for the response curve. To provide comparison with prior studies, we also measured the magnitude of amygdala activation by extracting percent signal change values for the familiar and novel face conditions using MarsBar [35].

Repeated measures ANOVAs were used to test for effects of temperament group (inhibited/uninhibited) and face condition (novel/familiar) on latency, duration, peak and magnitude variables (all alphas = .05). SAS statistical software (Version 9.1, SAS Institute Inc., Cary, NC) was used for statistical analyses.

#### Whole Brain Analysis

To explore temperament-based differences in the response to novelty across the whole brain, we used a second-level (random effects) general linear model analysis [36]. We compared responses between the two temperament groups for the contrast of novel > familiar faces. We used cluster-based methods to provide a whole-brain corrected significance threshold of *p *< .05. Based on simulations conducted with the AlphaSim module of AFNI http://afni.nimh.nih.gov/pub/dist/doc/manual/AlphaSim.pdf, a voxel p-value of .005 and contiguous cluster size of 40 controlled for family-wise error of .05 across the whole brain.

## Authors' contributions

JUB designed the study, collected and analyzed the data, and drafted the manuscript. SNA collected study data, performed clinical interviews, and revised the manuscript. RCS assisted with study design and revised the manuscript. DHZ assisted with study design and revised the manuscript. All authors have read and approved the final manuscript.
